# Effects of hypertension on subcortical nucleus morphological alternations in patients with type 2 diabetes

**DOI:** 10.3389/fendo.2023.1201281

**Published:** 2023-09-13

**Authors:** Feng Cui, Zhi-Qiang Ouyang, Yi-Zhen Zeng, Bing-Bing Ling, Li Shi, Yun Zhu, He-Yi Gu, Wan-Lin Jiang, Ting Zhou, Xue-Jin Sun, Dan Han, Yi Lu

**Affiliations:** ^1^Department of Medical Imaging, Laboratory of Brain Function, First Affiliated Hospital of Kunming Medical University, Kunming, Yunnan, China; ^2^Department of Endocrinology, First Affiliated Hospital of Kunming Medical University, Kunming, Yunnan, China

**Keywords:** type 2 diabetes mellitus, hypertension, volume analysis, shape analysis, subcortical nucleus

## Abstract

**Objectives:**

Type 2 diabetes mellitus(T2DM) and hypertension(HTN) are common comorbidities, and known to affect the brain. However, little is known about the effects of the coexisting HTN on brain in T2DM patients. So we aim to investigate the impact of HTN on the subcortical nucleus morphological alternations in T2DM patients.

**Materials & methods:**

This work was registered by the clinicaltrials.gov (grant number NCT03564431). We recruited a total of 92 participants, comprising 36 only T2DM patients, 28 T2DM patients with HTN(T2DMH) and 28 healthy controls(HCs) in our study. All clinical indicators were assessed and brain image data was collected for each participant. Voxel-based morphometry(VBM), automatic volume and vertex-based shape analyses were used to determine the subcortical nucleus alternations from each participant’s 3D-T1 brain images and evaluate the relationship between the alternations and clinical indicators.

**Results:**

T2DMH patients exhibited volumetric reduction and morphological alterations in thalamus compared to T2DM patients, whereas T2DM patients did not demonstrate any significant subcortical alterations compared to HCs. Furthermore, negative correlations have been found between thalamic alternations and the duration of HTN in T2DMH patients.

**Conclusion:**

Our results revealed that HTN may exacerbate subcortical nucleus alternations in T2DM patients, which highlighted the importance of HTN management in T2DM patients to prevent further damage to the brain health.

## 1 Introduction

Diabetes mellitus (DM) is a chronic and non-infectious disease that has seen a significant increase in incidence over recent years, with the International Diabetes Federation (IDF) reporting a 16% rise in the number of DM patients over the past two years (1). Alongside the classical clinical symptoms commonly associated with DM, including polydipsia, polyuria and weight loss, additional studies on brain health have revealed clinical manifestations of cognitive decline in individuals with DM, such as reduced executive function or decision-making ability. This cognitive impairment is particularly prevalent among individuals with T2DM, which constitutes around 90% of all DM cases (2).

In fact, abnormal brain structures are often responsible for cognitive impairment with clinical symptoms. Prior investigations have observed volume reduction in multiple cortical and/or subcortical regions in individuals with T2DM (3, 4). Although some studies have reported inconsistent findings, most have cited atrophy in the prefrontal cortex (5, 6) and medial temporal cortex, particularly in the hippocampus (6, 7). Furthermore, functional magnetic resonance imaging (fMRI) studies have uncovered a significant decrease in functional connections within the hippocampus of T2DM patients (8). Thus, both structural and functional abnormalities in these brain regions may play a role in the development and manifestation of cognitive impairment among T2DM patients.

Currently, the potential mechanisms underlying the subcortical gray matter alterations in T2DM patients are gradually becoming clear. Longitudinal studies with autopsy confirmation have established a link between T2DM and cardiovascular and cerebrovascular disease (CVD) (9), while vascular risk factors have been identified as important contributors to brain defects in T2DM patients (10, 11). HTN, in particular, has emerged as a significant factor, with approximately 75% of self-reported T2DM patients being afflicted by this condition (12). Indeed, HTN has been shown to independently affect brain structures, and the longer duration of HTN is associated with the smaller whole brain volumes (13). Moreover, a longitudinal study of middle-aged HTN patients has revealed thinning of the insular, frontal and temporal cortex in those with long-term exposure to this condition (14). In young HTN patients, cumulative systolic blood pressure (SBP) exposure has been negatively correlated with the morphological changes in subcortical regions such as the putamen, nucleus accumbens, pallidum, and thalamus (15). Importantly, structural abnormalities in several brain regions observed in HTN patients, including the frontal temporal cortex and basal nucleus, are also evident in T2DM patients. However, it remains unclear whether these defects are caused by HTN, T2DM, or their related complications. Furthermore, most previous T2DM studies have failed to regulate these factors, resulting in potentially biased outcomes. Only a recent study has examined the effect of HTN on cortex alterations in T2DM patients, confirming that coexisting HTN accelerates the reduction in cortex thickness, with a significant association between thinner cortex and cognitive decline in these patients (16). However, cognitive impairment may not be restricted to defects in the cortex alone, with other studies identifying associations between cognition and subcortical nucleus defects such as the thalamus (17), caudate nucleus (18), putamen (19), and hippocampus (20, 21). We, therefore, suspect that coexisting HTN may also impact subcortical nucleus, which could have implications for the cognitive function of T2DM patients.

Our study aims to investigate whether concurrent HTN exacerbates subcortical nuclei abnormalities in T2DM patients. VBM, automatic volume and vertex-based analyses (22) will be utilized to precisely locate and visualize subcortical nuclei alternations. Furthermore, we will evaluate the correlation between clinical features and subcortical nuclei structural abnormalities using neuropsychological tests and relevant clinical indicators.

## 2 Materials and methods

### 2.1 Participants

This was a prospective study, and the ethics committee of the First Affiliated Hospital of Kunming Medical University had approved the study protocol. In this study, 28 T2DM patients and 36 T2DM patients with HTN were recruited by the Department of Endocrinology at the First Affiliated Hospital of Kunming Medical University between June 2021 and March 2022. According to the 2010 edition of American Diabetes Association guidelines for diagnosis and treatment, T2DM was diagnosed by two experienced endocrinologists. The diagnosis of HTN was based on the criteria of 1999 World Health Organization-International Society of Hypertension Guidelines for the management of HTN. Blood pressure (BP) was recorded as the average of all measurements collected by a 24-hour ambulatory BP monitor. The exclusion criteria for T2DM were as follow: (a) Secondary diabetes and chronic diabetic complications such as clinical diabetic nephropathy, proliferative diabetic retinopathy, painful or symptomatic diabetic neuropathy (b) Mental disorders such as depression, epilepsy, Parkinson’s disease or schizophrenia (c) Previous central nervous system (CNS) injuries such as cerebral infarction, cerebral hemorrhage, brain tumor and brain trauma (d) Abuse of alcohol, drug addiction or other psychoactive agents. Besides, a total of 28 healthy controls (HCs), matched for age, gender and number of years of education, were recruited at the same time. They were also interviewed to affirm that there was no history of psychiatric illness, brain injury or drug abuse. All the participants were right-handed and provided signed informed consent.

All subjects’ blood samples were collected in the morning after fasting more than 10 hours overnight. General demographic and biomedical data of each participant were recorded by standard measurement method before magnetic resonance imaging.

The sample size was defined on the basis of results of a “*Post hoc*” power analysis, computed with G Power 3.1 [Parameters: effect size f(U) = 0.4; α err prob = 0.05; power (1-β err prob) = 0.93]. And output of the analysis revealed that a group sample size of at least 28 patients would have a 93% power to detect such a difference as statistically significant at a level (α) of 0.05 in the present study.

### 2.2 Neuropsychological assessments

All participants underwent a series of neuropsychological tests, including Mini Mental-Status Examination (MMSE) (23), Digital Symbol Substitution Test (DSST) (24), Hamilton Depression Scale (HAMD) (25) and Hamilton Anxiety Scale (HAMA) (26). The MMSE was used to assess general cognitive function of each participant. The DSST was involved in assessing the advanced cognitive functions, including executive function, attention and information processing speed. The HAMD and HAMA were used to evaluate the depression and anxiety of each subject. All neuropsychological tests were administered to all participants by the same and experienced psychiatric professional.

### 2.3 Data acquisition

All participants were scanned on a 3T Trio MRI system (GE Discovery 750w 3.0T) equipped with a 32-channel phase-array head coil. All participants were requested to remain calm, keep their eyes closed and avoid any movement during the image acquisition. Axial T1/T2-weighted images and T2 fluid attenuated inversion recovery (T2-FLAIR) images were performed to eliminate significant structural abnormalities of each subject. A high-resolution 3D fast-spoiled gradient recalled acquisition (FSPGR) sequence was acquired with the following parameters: rotation time (TR)/echo time (TE): 8.7/3.2ms, slice thickness: 1.0 mm, field of view (FOV): 256 mm × 256 mm, matrix size: 256 × 256, flip angle: 12°, slice number: 160 with no gap, and scan duration: 4 minutes 23 seconds. All sections were acquired parallel to the anterior–posterior commissure line.

### 2.4 Voxel-based morphometry analysis

Structural data of all subjects were processed by FMRIB Software Library (FSL, version5.0, http://www.fmrib.ox.ac.uk/fsl). A voxel-based morphometry (VBM) analysis in FSL was simplified to four steps: Brain Extraction, Tissue Segmentation, Spatial Normalization, Modulation and Smoothing. In the third step, we chose the non-linear registration instead of the linear registration to normalize our data. Especially, in the fourth step, we chose the 3mm sigma of isotropic Gaussian kernel for the smoothing of all grey matter images. Other steps were performed with the default options.

### 2.5 Subcortical grey matter volumetric analysis

Each subject’s 3D-FSPGR images was automatically segmented to the amygdala, accumbens, caudate, pallidum, putamen nucleus, hippocampus and thalamus by FMRIB’s Integrated Registration and Segmentation Tool (FIRST) (22), part of the FSL. FIRST was a deformable Active Appearance Model (AAM) based on a Bayesian framework, significantly different from VBM, it depended directly on the geometry/location of the structure boundary, not on the classification or smoothing extents of the tissue type. Because the quality of the automatic segmentation would affect the subsequent vertex-based analysis and statistical results, each step should be carefully examined, and corrected if necessary.

Meanwhile, the original volume of each subcortical nucleus would be obtained automatically at this stage. In order to eliminate the difference in head size among subjects, the brain image extracted from the single whole-head input data, and non-linear registered to the MNI152 space, which used for normalization of head size (27).

### 2.6 Shape analysis of subcortical grey matter

Vertex-based shape analysis was performed after the automatic segmentation. In this stage, the first step was to perform two-stage linear registration of the training data to achieve a more robust and accurate pre-alignment of the subcortical structure. Then a probabilistic math model was trained to collect the shape and intensity parameters of the training data. When applied to the new data, the math model is registered to the native space using the inverse transform, and then vertex analysis was performed by carrying out a multivariate test on the three-dimensional coordinates of corresponding vertices (22). Each vertex was analyzed independently to check the difference in the three-dimensional parameters. These differences are visualized by subsequent statistical analysis.

### 2.7 Statistical analysis

All demographic, biomedical and neuropsychological data tests were processed in SPSS, version 26.0 (SPSS, Chicago, IL, USA). An unpaired two-sample t-test was performed to continuous variables in this study. Moreover, a chi-square test was using for categorical variables and a nonparametric Mann–Whitney U test for non-normally distributed variables. All tests were 2-sided, and a ***p*** < 0.05 was considered statistically significant. Continuous variables were expressed as mean ± standard deviation (SD), and as medians ± interquartile ranges (IQR) for other variables.

In this study, the effect of T2DM and T2DMH on the subcortical nucleus was evaluated separately. Firstly, in order to access the volume difference between the T2DM and HCs, an analysis of covariance (ANCOVA) was conducted for the normalized volume data of each structure (28). Age, gender and the duration of DM were regarded as covariates and a ***p*** < 0.05 was considered statistically significant. Meanwhile, VBM and vertex-based analyses were respectively used to access the morphometry and shape difference of each structure between the T2DM and HCs in FSL. Similarly, age, gender and the duration of DM were regarded as covariates again. Clusters were identified using a threshold-free cluster enhancement (TFCE) by running 5000 random permutations. The cluster-wise threshold was setting at ***p*** < 0.05 with a family wise error (FWE) correction. Subsequently, the same step applied between the T2DM and T2DMH.

Correlations between the volume/shape difference and the cognitive impairment or related clinical characteristics were performed with a partial correlation analysis. The volume difference-based correlation analysis was accessed in SPSS. Different from the volume analysis, the shape difference -based correlation analysis was calculated in Permutation Analysis of Linear Model (PALM, https://fsl.fmrib.ox.ac.uk/fsl/fslwiki/PALM) (28), which was a tool that allowed inference using permutation methods, offering a number of features not available in other analysis software. ***p*** value was still corrected by FWE.

## 3 Results

### 3.1 Demographic, biomedical and neuropsychological characteristics

The demographic biomedical and neuropsychological characteristics of all subjects were shown in **Table 1**. There was no significant difference in demographic characteristics between groups (***p >*** 0.05) except for a higher average age in the T2DMH than the T2DM (*p* = 0.005). However, in the comparison of biological and neuropsychiatric characteristics, multiple indicators were statistically different among groups. Compared with the HCs, several characteristics including Your 10-year risk (*p* < 0.001), vascular age (*p* < 0.001), TG (*p* = 0.009), FPG (*p* < 0.001) and MMSE (*p* = 0.004) were significantly increased in the T2DM and several other indicators including ALT(*p* = 0.025), AST(*p* < 0.001), HDL(*p* < 0.001), INS(0) (*p* = 0.028), HOMA beta(*p* < 0.001) and DSST(*p* =0.001) were significantly decreased in the T2DM. Moreover, multiple indicators including the duration of DM (*p* = 0.038), Your 10-year risk (*p* < 0.001), vascular age (*p* < 0.001), SBP (*p* < 0.001), DBP (*p* < 0.001), FI (*p* < 0.001) and HOMA-IR (*p* = 0.036) were observed with a higher level in the T2DMH than the T2DM. No decreased characteristics were observed in the T2DMH contrast to the T2DM.

**Table 1:** Characteristics of study participants.

Characteristics	HCs (n=28)	T2DM (n=36)			T2DMH (n=28)		T2DM-HCs		T2DMH-T2DM	
							t/χ2	*P*	t/χ2	*P*
Demographic characteristics										
Age, mean (SD),years	50.46 (6.374)	49.19 (5.879)			54.39 (7.87)		-0.826	0.412	2.920	0.005*
Sex (Male/Female)	26/10	15/13			18/10		2.38	0.189	0.462	0.341
Education level, mean (SD),year	10.86 (3.932)	11.00 (3.381)			11.14 (2.84)		0.156	0.876	0.180	0.858
BMI, mean (SD), kg/m^2^	23.70 (2.52)	24.27 (2.56)			25.15 (2.19)		0.893	0.376	1.336	0.187
Hand (left/right)	0/28	0/36			0/28		–	–	–	–
Diabetes-related clinical characteristics										
Time since diagnosis of T2DM, mean (SD), years	–	5.31 (5.21)			8.43 (6.59)		–	–	2.117	0.038*
HbA_1c_,mean (SD), % (mmol/mol)	–	9.13 (2.44)			8.59 (1.97)		–	–	-0.966	0.338
FPG, mean (SD), mmol/l	4.87 (0.37)	7.63 (3.41)			6.93 (3.03)		4.823	<0.001*	-0.854	0.357
FI, mean (SD), pmol/l	10.93 (7.70)	7.64 (3.80)			11.92 (4.94)		-2.244	0.028*	3.929	<0.001*
HOMA-IR, mean (SD)	2.43 (2.05)	2.67 (1.85)			4.04 (2.92)		0.498	0.620	2.170	0.036*
HOMA-beta, mean (SD),%	162.19 (74.56)	55.97 (44.07)			85.08 (102.58)		-7.109	<0.001*	1.533	0.130
LnHOMA-IR	0.75 (0.43)	0.71 (0.85)			1.16 (0.65)		-0.251	0.803	2.294	0.025*
LnHOMA-beta	5.01 (0.39)	3.71 (0.84)			4.30 (0.66)		-8.215	<0.001*	3.082	0.003*
Blood Pressure										
Time since diagnosis of HTN, mean (SD), years	–	–			6.79 (5.36)		–	–	–	–
Systolic, mean (SD), mmHg	117.18 (12.51)	114.83 (12.79)			132.25 (13.29)		-0.735	0.465	5.313	<0.001*
Diastolic, mean (SD), mmHg	77.36 (9.67)	74.75 (7.65)			82.46 (7.60)		-1.205	0.233	4.013	<0.001*
Vascular risks										
Your 10-year risk (%)	9.55 (7.35)	18.29 (9.93)			34.23 (16.79)		4.05	<0.001*	4.453	<0.001*
Vascular age, year	54.21 (10.08)	66.58 (12.75)			80.86 (6.08)		4.209	<0.001*	5.909	<0.001*
IMT, mean (SD), mm	–	0.87 (0.17)			0.94 (0.22)		–	–		
Lipid profiles										
TC, mean (SD), mmol/l	4.82 (0.67)	4.60 (1.06)			4.29 (0.92)		-0.95	0.346	-1.216	0.229
TG, mean (SD), mmol/l	1.61 (0.77)	2.59 (1.82)			4.60 (1.06)		2.926	0.005*	-0.173	0.863
LDL-c, mean (SD), mmol/l	1.35 (0.56)	2.90 (0.85)			2.68 (0.72)		8.722	<0.001*	-1.087	0.281
HDL-c, mean (SD), mmol/l	3.27 (0.93)	1.04 (0.25)			1.02 (0.27)		-12.407	<0.001*	-0.245	0.808
Renal function										
BUN, mean (SD), mmol/l	4.52 (0.97)	4.41 (1.06)			4.54 (1.15)		-0.411	0.683	0.437	0.663
Cr, mean (SD), μmol/L	73.81 (12.43)	71.02 (16.57)			70.05 (9.93)		-0.744	0.460	-0.275	0.784
UA, mean (SD), μmol/L	308.12 (85.09)	328.89 (64.07)			345.68 (92.14)		1.115	0.269	-0.859	0.394
UAER, mean (SD),mg/24h	–	35.27 (38.06)			88.78 (235.31)		–	–	1.345	0.184
UPCR, mean (SD), mg/24h	–	51.72 (101.43)			206.38 (804.05)		–	–	1.145	0.257
Cognitive assessments										
MMSE, mean (SD)	27.11 (3.25)	29.28 (2.59)			28.71 (3.37)		2.892	0.006*	-0.757	0.452
DSST, mean (SD)	45.86 (21.43)	32.18 (10.27)			29.39 (11.41)		-3.111	0.004*	-1.026	0.309
HAMD, mean (SD)	3.89 (3.93)	3.81 (3.27)			4.11 (2.99)		-0.097	0.923	0.380	0.705
HAMA, mean (SD)	3.54 (4.33)	4.61 (3.24)			4.82 (2.67)		1.137	0.260	0.278	0.782

HCs, healthy controls; T2DM, Type 2 diabetes mellitus; T2DMH, T2DM patients with hypertension; BMI, body mass index; H bA1c, the levels of Hemoglobin A1c; FPG, Fasting plasma glucose; FI, Fasting insulin; HOMA-IR, homeostatic model assessment of insulin resistance; HOMA-beta, homeostatic model assessment of insulin Beta-cell function index; LnHOMA-IR and LnHOMA-beta were the natural logarithms of HOMA-IR and HOMA-beta. TC, Total cholesterol; TG, Triacylglycerol; LDL-c, low-density lipoprotein cholesterol; HDL-c, high-density lipoprotein cholesterol; IMT, Intima-media thickness; BUN, blood urea nitrogen; Cr, Creatinine; UA, Uric acid; UAER, Urinary albumin excretion rates; UPCR, Urine protein to creatinine ratio; MMSE, Mini-Mental State Examination; DSST, Digit Symbol Substitution Test; HAMD, Hamilton depression scale; HAMA, Hamilton Anxiety Scale. *: P value was less than 0.05, which is statistically significant.

The symbol "-" represents parameters that were not applicable or no significance for measurement in the column.

The bold values represent P-values with statistical differences.

### 3.2 Voxel-based morphometry analysis

Among T2DM, T2DMH and HC groups, there was no significant alternations in grey matter found by VBM analysis, at the threshold of *p* < 0.05(FWE-corrected).

### 3.3 Volumetric analysis of subcortical gray matter

Statistical results for normalized volumes of the 14 subcortical nucleus between groups had been showed in **Table 2**. An ANCOVA revealed that there was no significant difference was found in all subcortical nucleus volume between the T2DM patients and HCs. However, compare to T2DM, the volume of right thalamus in T2DMH patients showed a significant reduction (F= 4.555, *p*=0.037). Meanwhile, correlation analysis showed that the reduction of right thalamic volume in T2DMH patients was negatively correlated with the duration of HTN (*p* =0.002, r=0.40). **Table 3**.

**Table 2:** Normalized subcortical grey matter structural volumes of study participants (mm^3^).

Structures	HCs (n=28)	T2DM (n=36)	T2DMH (n=28)	T2DM-HCs		T2DMH-T2DM	
				F	*P*	F	*P*
Left							
Accu	462.75 ± 108.702	483.44 ± 89.395	456.64 ± 92.206	0.179	0.674	0.316	0.576
Amyg	1073.21 ± 245.512	1122.22 ± 217.149	1158.93 ± 278.196	0.013	0.910	0.733	0.396
Caud	3351.39 ± 379.624	3401.75 ± 389.437	3286.96 ± 423.778	0.011	0.917	0.374	0.543
Hipp	3439.61 ± 364.640	3589.72 ± 408.713	3531.11 ± 589.248	0.877	0.353	0.033	0.856
Pall	1846.71 ± 409.993	1820.33 ± 252.956	1808.89 ± 324.462	0.037	0.847	0.479	0.452
Puta	4955.29 ± 604.757	5039.08 ± 560.724	4836.39 ± 540.755	0.056	0.814	0.197	0.659
Thal	7822.11 ± 702.907	7997.22 ± 639.488	7584.11 ± 675.678	0.082	0.775	1.322	0.255
Right							
Accu	363.82 ± 97.604	354.64 ± 89.126	344.82 ± 93.281	0.212	0.647	0.004	0.947
Amyg	1104.25 ± 383.398	1072.28 ± 197.825	1147.50 ± 241.613	0.853	0.359	0.631	0.430
Caud	3499.61 ± 372.473	3543.17 ± 420.635	3419.25 ± 397.934	0.156	0.694	0.410	0.524
Hipp	3584.71 ± 428.424	3753.08 ± 391.165	3759.43 ± 496.421	1.145	0.289	0.782	0.380
Pall	1851.96 ± 339.667	1804.14 ± 201.035	1843.57 ± 337.670	0.403	0.528	0.001	0.978
Puta	4909.64 ± 554.494	4960.89 ± 489.745	4761.68 ± 508.780	0.172	0.680	0.187	0.667
Thal	7556.61 ± 632.588	7775.56 ± 535.139	7305.82 ± 641.500	0.672	0.416	3.632	0.042*

Accu, Accumbens; Amyg, Amygdala; Caud, Caudate; Hipp, Hippocampus; Pall, Pallidum; Puta, Putamen; Thal, Thalamus

*: P value was less than 0.05, which was statistically significant.

The bold values represent P-values with statistical differences.

**Table 3:** Correlation results for the subcortical GM in volume and shape analysis.

Measures	cluster	r/R^2^	*P*
Correlation with the duration of HTN			
The volume of right thalamus	–	-0.40	0.002*
The shape of left thalamus	87	-0.43	0.002*
The shape of right thalamus	181	-0.46	0.003*
	262	-0.46	0.003*

*: P value was less than 0.05, which was statistically significant.

The symbol "-" represents parameters that were not applicable or no significance for measurement in the column.

The bold values represent P-values with statistical differences.

### 3.4 Shape analysis of subcortical gray matter

**Figure 1:**
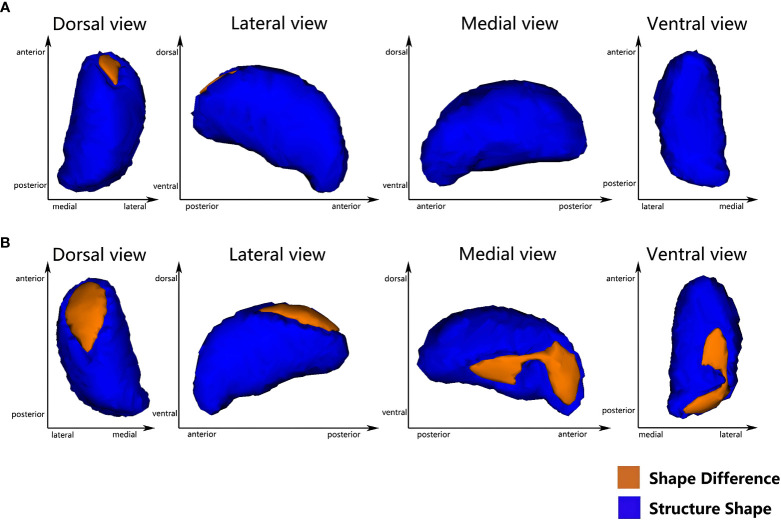
Vertex-wise comparison between T2DMH and T2DM shows significant regional shape deformation on the dorsal and medial aspects of the left thalamus **(A)**, and the dorsal, medial and ventral aspects of the right thalamus **(B)** (FWE-corrected, *p*<0.05).

**Figure 2:**
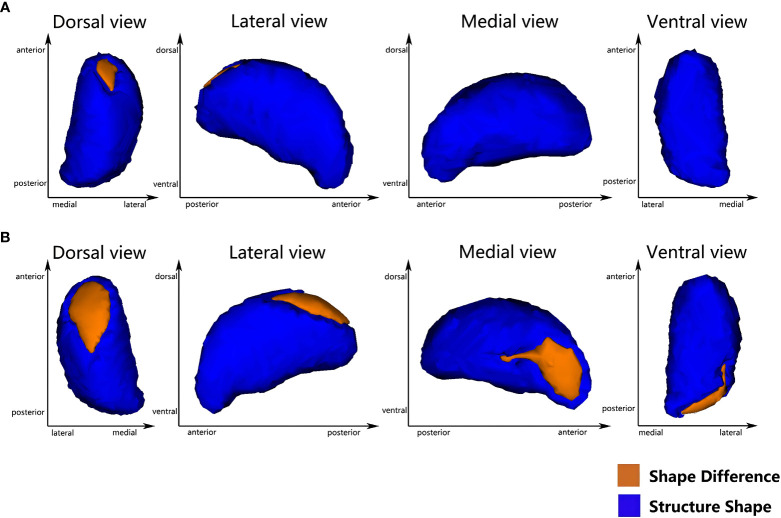
Vertex-wise comparison shows a significant negative correlation between the duration of HTN and the regional shape deformations on the dorsal and medial side of the bilateral thalamus in T2DMH patients **(A)** the left thalamus; **(B)** the right thalamus) (FWE-corrected, *p <*0.05).

Vertex-based shape analysis revealed significant regional shape deformation on the medial, dorsal aspects of the left thalamus (cluster_a_, voxels=87, *p*=0.036, FWE-corrected, x=-9, y=-5, z=11) and the medial, dorsal, ventral aspects of the right thalamus (cluster_b_, voxels=283, *p*=0.014, FWE-corrected, x=13, y=-33, z=0; cluster_c_, voxels=287, *p*=0.008, FWE-corrected, x=14, y=-8, z=14), which were shown in **Figure 1**. No significant difference was found in the subcortical structures between the T2DM and HCs (*p* > 0.05, FWE-corrected). Meanwhile, there were significant negative correlations of the duration of HTN with regional shape deformation of the left and right thalamus (left thalamus, cluster_a_, voxels=87, *p*=0.002, FWE-corrected, x=-11, y=-5, z=13; right thalamus, cluster_b_, voxels=181, *p*=0.003, FWE-corrected, x=12, y=-33, z=1; cluster_c_, voxels=262 *p*=0.003, FWE-corrected, x=14, y=-6, z=12, r=0.46), which were shown in **Figure 2** and **Table 3**.

## 4 Discussion

In this study, we exploited the VBM, automatic volume and vertex-based shape analyses to determine the subcortical nucleus abnormalities in T2DM and T2DMH patients. Our results showed that there were no significant alterations in the subcortical nucleus of T2DM patients compared with HCs. However, we observed a volume reduction in the right thalamus and the regional shape deformation in the bilateral thalamus of T2DMH patients when comparing T2DM patients. A vertex-based shape analysis revealed additional abnormalities of the thalamus in T2DMH patients than the VBM and automatic volume analysis. Furthermore, we found negative correlations between all the volume/shape alternations in the thalamus and the duration of HTN.

The present study did not show any significant subcortical nucleus defects in T2DM patients when compared to HCs, which differed from the consistent findings in previous studies that emphasized the hippocampal atrophy (6, 7). We hypothesized that blood pressure control could be the primary factor explaining our results. A recent study found that the presence of HTN in T2DM patients contributed to a further reduction in cortical thickness (16). Additionally, HTN alone had been linked to an increased risk of hippocampal atrophy (29). It was evident that in most previous T2DM studies, blood pressure was not adequately controlled in all participants, which may have contributed to inconsistent results. Therefore, obtaining a more reliable conclusion may necessitate balancing the blood pressure profile in future studies. Secondly, recent evidence suggested a compensatory mechanism within the hippocampus that may compensate for structural defects in the early stage of T2DM or before cognitive impairment develops (30). Meanwhile, similar studies also have confirmed that hippocampal atrophy was more likely to occur in T2DM patients with cognitive impairment (31). Thus, based on our present data, we speculate that the compensation mechanism may have played a role in this study and may explain why hippocampal defects were not observed. It is necessary to conduct more longitudinal studies with larger sample sizes to support our hypothesis in the future.

An important finding of this study was that T2DMH patients exhibited a volume reduction in right thalamus and the regional deformation in the bilateral thalamus compared to T2DM patients. Obviously, the vertex-based shape analysis could unveil nuances that VBM remained elusive, owing to the robust mathematical framework afforded by FIRST (22). Thalamus was considered to be an important structure for the generation and transmission of high-level neural activities, all kinds of sensory conduction of the whole body except for the sensation of smell were transformed in the thalamus, and then projected to the cerebral cortex to produce specific sensation (32). In addition, thalamus was thought to be associated with learning memory and executive function (33). So the atrophy of the thalamus in T2DMH patients may imply a loss of neurons, which may result in increased risks of sensory or cognitive decline. In fact, the thalamus may have shown the metabolic or functional defects before the obvious structural abnormalities. A previous Magnetic Resonance Spectroscopy (MRS) research found that the N-Acetyl-Aspartate/Creatin (NAA/Cr) of the bilateral thalamus was reduced in HTN patients (34). As a unique metabolite of neurons, the reduction of the NAA level meant that the activity of neurons was reduced, and it was related to the cognitive level. Combined with our research, it showed that HTN was an important risk factor for thalamus defects in T2DM. Although the exact physiological or pathological mechanism of the thalamus defects which was dominated by HTN in T2DM patients was still unclear, some robust researches had shown that the oxidative stress dominated the vascular damage in both HTN and T2DM, including large blood vessels and microcirculation (35, 36). The oxygen free radicals which were produced in the process of oxidative stress have been proved to be an important factor which led to the thalamus degeneration (37). Therefore, we speculated that the oxygen free radicals may enter the thalamus through the microcirculation and accumulate constantly, thus these overloaded oxygen free radicals created an environment which was unfavorable to the survival of thalamic cells. Over time, a neurodegeneration may occur in thalamus which was exposed to this environment. In addition, inflammatory factors related to the oxidative stress may also participate in the pathology of thalamus defects (38). These assumptions may provide some insights for explaining thalamus defects, more rigorous and direct evidence was needed to show the relationship between the oxidative stress and thalamus defects of T2DMH in the future.

Furthermore, our study revealed that the morphological deformation in T2DMH patients were predominantly localized in the medial and dorsal regions of the left thalamus, as well as the medial, dorsal, and ventral regions of the right thalamus. It is worth noting that the dorsal and medial thalamus had been shown to be structurally and functionally connected to the prefrontal and temporal cortex, while the ventral thalamus was associated with the parietal cortex (32). Previous research has suggested that subcortical nucleus neural degeneration was frequently a secondary effect of defects in the cortex regions which were synaptically associated with corresponding subcortical nucleus (39). Therefore, we proposed that the deformation observed in the dorsal, medial, and ventral regions of the thalamus may be associated with structural defects in the corresponding cortex regions. Previous researches on T2DM patients that did not control their blood pressure had already observed defects in the prefrontal (5, 16), temporal, and parietal cortex (40). Nevertheless, our current study suggested that the presence of HTN may be a crucial factor in contributing to cortex defects in T2DM patients. It has been reported that HTN may impair the blood supply system of cerebral cortex by altering cerebral vascular microcirculation hemodynamics (35), which may lead to the prolonged cortical ischemia and cortex atrophy (41). An arterial spin labeling (ASL) study had revealed that the decrease of cerebral blood flow in HTN patients were primarily concentrated in the frontal, temporal, and parietal lobes (42). Therefore, we speculated that the prefrontal, temporal, and parietal cortex atrophy may lead to the neural degeneration in the dorsal, medial, and ventral regions of the thalamus via a cortical-subcortical synapse connection. Currently, we hypothesize that concurrent HTN may exacerbate brain damage and participate in cortex defects by influencing the thalamus deformation in T2DMH patients.

Moreover, another important finding in our study was the thalamic volume reduction and deformation were negatively correlated with the duration of HTN in T2DMH patients, which suggested that the longer the duration of HTN, the more significant subcortical nucleus defects in T2DMH patients. Our finding was consistent with previous studies that had shown a correlation between the duration of HTN and brain volume (13). Meanwhile, a longitudinal study of HTN patients had emphasized the relationship between the duration of HTN and the incidence of T2DM (43). This indicated that the influence of HTN on T2DM may exist before the confirmed diagnosis of T2DM. Integrating these findings suggested that the effects of HTN on T2DM had increased gradually with the duration of HTN. Effective interventions, such as blood pressure screening and follow-up treatment for early T2DM patients, were necessary to reduce the potential threat of HTN to brain health.

For cognition, we found that the MMSE score of T2DM group was higher than that of HCs, and the score was higher in the T2DMH group compared to the T2DM group. The outcome, at first glance, appeared paradoxical. However, a more nuanced understanding emerged when we turned our attention to those participants endowed with the levels of education. There is a higher education level of T2DM and T2DMH participants in two controls. In this context, the facile nature of the MMSE test appeared to veil the cognitive decline evident in these patients, as their scores harmoniously nestled within the bounds of normalcy. In addition, our study unveiled the potency of the DSST test in discerning cognitive decline in the T2DM patients. However, the DSST scores exhibited a convergence between the T2DM and T2DMH. This result may imply that the DSST, despite its utility as an indicator of glycemic control-related cognition, might not possess the requisite sensitivity to detect the concomitant presence of HTN. This intriguing observation prompts consideration that the absence of correlation between thalamus alterations and DSST scores in T2DMH patients could be attributed to this potential limitation.

Although the current study did not establish a clear relationship between subcortical nucleus defects and cognitive impairment in T2DMH patients, animal models had demonstrated the impact of the mediodorsal thalamus-prefrontal cortex loop on cognition (44, 45). According to this framework, the mediodorsal thalamus dominated the cortex representation under different behavioral conditions, and was associated with cognitive impairment. We speculated that an insufficient sample size and indistinguishable cognitive scores between T2DM and T2DMH patients in this study may limit us to describe the accurate relationship between subcortical nucleus defects and cognitive impairment in T2DMH patients. Meanwhile, it was noting that these animal studies had also confirmed the findings of past decades brain imaging researches which revealed that prefrontal cortex may more strongly associated with cognition (46). For example, this relationship was proved in different cohort population involving depression, schizophrenia, and Alzheimer’s disease (47–49). Given the evidence from these robust studies, it was plausible to postulate that while subcortical nucleus defects may exert some influence on cognition, the relationship between cortical defects and cognitive impairment in T2DMH patients appeared to be more straightforward.

The present study had some limitations that should be acknowledged. Firstly, this study was a horizontal study with a relatively small sample size, so the conclusion cannot be extended to the general population. Secondly, the use of medications for T2DM or T2DMH subjects could potentially impact our findings to some extent. Meanwhile, this study employed the MMSE and DSST, two simple cognitive function screening scales, to evaluate the cognition of all participants. Future researches should consider more comprehensive cognitive evaluation methods to investigate the potential relationship between subcortical nucleus defects and cognition in T2DMH patients. Lastly, although we speculated on the relationship between cortex and subcortical nucleus defects in T2DMH based on related literature, we did not separately analyze the cortex in the current study, and thus could not further explore this relationship.

## 5 Conclusion

In summary, our study suggested that HTN may exacerbated brain damage in T2DM patients and may potentially threaten their cognitive abilities. Thus, early interventions such as an effective blood pressure control could mitigate extra brain damage in T2DMH patients. Regrettably, our findings did not yield conclusive evidence to establish a direct association between subcortical nucleus deficits and cognition in T2DMH patients. Further longitudinal researches or more comprehensive cognition investigations may provide valuable insights into this inquiry.

## Data availability statement

The raw data supporting the conclusions of this article will be made available by the authors, without undue reservation.

## Ethics statement

The studies involving humans were approved by the ethics committee of the First Affiliated Hospital of Kunming Medical University. The studies were conducted in accordance with the local legislation and institutional requirements. The participants provided their written informed consent to participate in this study.

## Author contributions

LS and YL designed the study and experiments. FC, Z-QO, Y-ZZ, and B-BL contributed to the drafting of the manuscript. FC and YL contributed to the data analysis. YL and X-JS edited the manuscript. Y-ZZ and YL acquired the magnetic resonance images. YZ, H-YG, W-LJ, TZ, X-JS, and DH contributed to the clinical studies and data acquisition. All authors contributed to the article and approved the submitted version.
